# Competitive Exclusion between Piroplasmosis and Anaplasmosis Agents within Cattle

**DOI:** 10.1371/journal.ppat.0040007

**Published:** 2008-01-25

**Authors:** Loubna Dib, Idir Bitam, Maja Tahri, Mourad Bensouilah, Thierry De Meeûs

**Affiliations:** The Scripps Research Institute, United States of America

There are about 869 species of ticks described so far [1], most of which are responsible for the transmission of a huge diversity of microorganisms belonging to almost all the trees of life (viruses, bacteria, protozoa, fungi, and nematodes) [2–4]. Despite the strong opportunity for interaction between these pathogenic species, little is known about the competitive interactions between tick-borne diseases within the vector and the vertebrate host (see [3] for review).

Severity of symptoms is often found associated with co-infection by different pathogens (e.g., [3,5–7]). Moreover, dual infections may affect the therapeutic strategies [5]. Exploring the incidence of co-infections and testing for possible interactions (positive or negative) thus represent important objectives.

Theileria annulata, Babesia bovis, and Anaplasma marginale are among the most economically important haemoparasitic tick-borne diseases of ruminants worldwide [8] and represent a serious economic challenge, particularly in developing countries [9–11]. In this paper we analyse the co-occurrence of these pathogens within individual bovine hosts in northeastern Algeria (North Africa) from one original dataset and one published dataset from the same region [12] that we have reanalysed, and test for the existence of positive or negative associations between these pathogens. We then discuss the implications of our findings in terms of therapeutic strategies and further studies.

All samples were collected around Boutheldja, a small town located on the northeast border of Algeria (close to Tunisia) and east of Annaba in Algeria (GPS coordinates 36° 45′ 7.0 N; 8° 10′ 0 E) corresponding to an area of 113.53 km^2^ (Figure S1). The sampling period extended from July to December 2004. Ill cattle were diagnosed by private veterinarians, who were contacted directly by their owners. The main criteria used for diagnosis were the presence of the ticks, a hyperthermic condition, presence of icterus, ganglionic hypertrophy, feebleness, lack of appetite, anorexia, anaemia, and dehydration. In case of positive diagnostic a blood smear was carried out, as described in Table 1. Parasites were then identified according to morphology, localisation in the erythrocytes, and using the key proposed by Morel in 1981 [13]. We also reanalysed the data from a published descriptive work [12] undertaken in 2002 in the same part of Algeria on 54 cattle with an identical approach as described above.

**Table 1:**
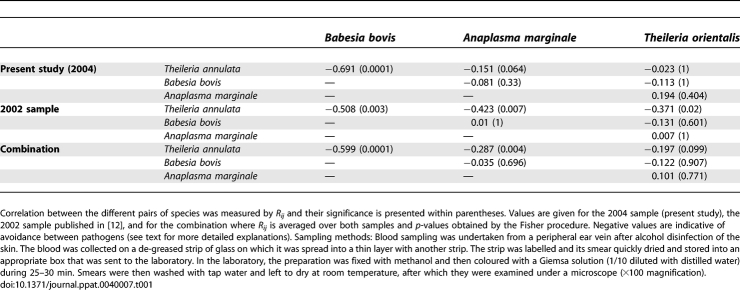
Within-Cattle Occurrence Correlation between Pathogens

Four species were identified in the samples: T. annulata, B. bovis, A. marginale, and Theileria orientalis. Note that *Anaplasma* are rickettsial bacteriae while the others are apicomplexan sporozoan (piroplasms), and that transovarial transmission is only known for Babesia sp. and not for the three other species. In the area investigated, Theileria sp. are transmitted by different *Hyaloma* ticks, B. bovis is transmitted by the tick Boophilus annulatus, and A. marginale by B. annulatus and Rhipicephalus bursa [4]. All are intracellular parasites [14].

In 2004, the most frequently encountered pathogenic agent was T. annulata (47.6% of infected cattle), followed by A. marginale (40.5%), B. bovis (33.3%), and T. orientalis (2.4%). In 2002, as presented by [12], some differences were observed with 74%, 24%, 8%, and 16% for T. annulata, A. marginale, B. bovis, and T. orientalis, respectively. The comparison of microbe prevalences between 2002 and 2004 samples was undertaken with an approximation of the Fisher exact test with 1,000,000 randomisations using the module Struc of Genepop version 3.1.c [15]. Randomisation tests gave significant differences (*p*-values ≤ 0.036) for all species but A. marginale (*p*-value = 0.117). In 2004 (original sample), 12 hosts appeared negative, 15 were infected by T. annulata only, ten by B. bovis only, seven by A. marginale, and four were infected both by B. bovis and *A. marginale*, five by T. annulata and *A. marginale*, and one by T. orientalis and A. marginale. In 2002 [12], four hosts appeared negative, 29 were infected by T. annulata only, three by B. bovis only, four by A. marginale, three by T. orientalis, and one was infected both by B. bovis and *A. marginale*, five by T. annulata and *A. marginale*, two by T. orientalis and A. marginale, and three by T. annulata and *T. orientalis*. Presence or absence of a particular micropathogen was treated as a phenotypic character. Thus there were four such phenotypic characters each taking the value 1 (absence) or 2 (presence). To study the association between these four characters a correlation coefficient, initially designed for genetic data, was adapted to our data. This coefficient of correlation is noted *R_ij_*, where *i* and *j* stands for the pair of loci (or as here, phenotypic characters), the association of which is under study. It was computed by Genetix 4.02 [16]. This parameter is described in a series of articles cited in the Genetix help menu (i.e., [17–19]). If *n*_11_ is the number of cattle without pathogens *i* or *j*, *n*_12_ the number of cattle infected by *j* but not by *i*, *n*_21_ those infected by *i* but not *j*, *n*_22_ those infected by both species, and *n* the total sample size, then for a given pathogen pair *ij*:

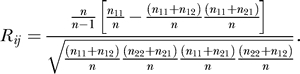



The significance of *R_ij_* was tested by a permutation procedure. Phenotypes (1 or 2) of the two characters studied (e.g., *T. annulata* and B. bovis) are associated at random a number of times (10,000) and a statistic *R* is recalculated on the randomised data set. The exact *p*-value is estimated as the proportion of statistics from randomised data sets that are larger or equal to the one observed in the real data set. This test was undertaken under Genetix 4.02, which uses Weir's *R* [20] as the statistic. However, given how we coded data, this statistic simply corresponds to *R_ij_* absolute value (bi-lateral testing). The 12 ill cows that were negative according to the cytological tests in 2004 were not taken into account for this study, considering that microbes might have been missed (false negative). The four negative cattle from [12] were also ignored. However, undertaking the same tests and including these data as true negatives (no microbe) did not change the results described below. To unite both data sets (2004 and 2002), we simply averaged *R_ij_* (unweighted mean) and combined *p*-values with the Fisher procedure [21] where the quantity 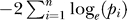 can be compared to a chi-square distribution with 2*n* degrees of freedom, with *n* the number of tests (here *n* = 2) and *p_i_* the *p*-value of the *i*th test. The results of the correlation study between pairs of pathogenic agents provided mainly negative correlations (five out of six for combined data, Table 1). For the original sample (2004), only one of the correlations, between T. annulata and B. bovis, was highly significant. In fact, no cattle individual was found co-infected by both pathogens despite an expected co-occurrence of 0.476*0.333*42 = 7 co-infected individuals. The second interesting result, though marginally non-significant, was observed between T. annulata and A. marginale, for which only five individuals were found co-infected for an expected frequency of height. All other pairs were not significant, but we can notice that with only one case reported for T. orientalis, the rarity of this species in 2004 makes any conclusion impossible for this pathogen. Nevertheless, the analysis combining 2002 [12] and 2004 samples confirms all the tendencies observed (Table 1). An absolute exclusion exists between T. annulata and B. bovis (no co-infection observed), a strong avoidance characterises the relationship between T. annulata and A. marginale, and a moderate one (if any) between this species and T. orientalis.

Because *R_ij_* is oriented and because we coded absence as 1 and presence as 2 for each phenotype, a positive value would bear witness to a positive association between the two pathogenic agents within the same individual hosts, while a negative value gives evidence for avoidance between the pairs of microbes studied. In the first case, different interpretations can be formulated as cooperation between pathogens (immunocompromising by one agent, opening the gate to infection by the other) or identical ecological needs (e.g., same vector). Immunosuppressive effects are what seem to be most of the time observed for various pathogenic agents, in particular in tick-borne diseases, as babesioses with other parasites in mice, or anaplasmosis with louping ill virus in sheep and goats (reviewed in [22]). In the case of a negative association, a hypothesis for competitive exclusion or at least a negative interference (cross-immunisation) can be advanced. This is the case for T. annulata and B. bovis, which appear to strictly avoid co-infection. This is also the case for T. annulata and A. marginale, though to a lower extent and probably also for T. annulata and *T. orientalis*, though the rarity of the latter species (low statistical power) forbids a definitive conclusion. This may seem to contradict the conclusions found in other studies on similar systems [23–25], though prevalence differences, lack of detailed data, and absence of specific analysis may be the main causes of this apparent discrepancy. Significant negative correlations always involve T. annulata against the other pathogen species. In the area studied, Theileria sp. and the other pathogens (B. bovis, A. marginale) are transmitted by different tick species [4]. Thus, competition must occur within the vertebrate host. Within-host competition between microbes (mostly intra-specific) was reviewed in Read and Taylor [26], with the best documented examples apparently found in Plasmodium sp. Competition was poorly studied in tick-borne diseases and only suspected once (to our knowledge) between Anaplasma phagocytophilum, Ehrlichia muris, and Babesia microti in unfed adults of Ixodes persulcatus in northwestern Russia [27]. This might confirm a generalised weak compatibility between these three kinds of intracellular pathogens within the vector [27] as well as within the vertebrate host (present study). Weak compatibility between these pathogens was never evidenced in vertebrate hosts for which specific studies are scarce or difficult to interpret in that perspective. Immunological surveys are not conclusive enough, as they may better reflect the history of sequential infections, not necessarily all successful, experienced by one host. Many studies report the co-occurrence of tick-borne micro-pathogens (e.g., [3,5,12,23,24] and references therein), and specific analyses seemed to conclude with positive associations or even suggested cooperation in multi-infected mice: between Borrelia burgdorferi (Lyme disease agent) and B. microti (transmission to tick is enhanced) [6] or between B. burgdorferi and A. phagocytophilum (*Borrelia* number is increased in co-infected mice) [28] (see also [29] for review and references therein). Synchronous infections with Babesia divergens and A. phagocytophila seem very common, but their interaction remains poorly understood and a suppression of *Babesia* by *Anaplasma* even seems possible (reviewed in [25]). As suggested above, such exclusion may be mediated by host immune system (concomitant immunity) or by direct interference (one pathogen tends to eliminate the other). Within the vector, most studies only report the co-occurrence of different microbes [10,29–37]. Associations between different pathogenic species were rarely tested, and only positive associations [6,28,38,39] or no effect [40] between borrelia spirochaetes and co-infecting microbes were detected.

Exclusion between pathogens may appear beneficial to the host, as severity of symptoms is often found associated with co-infection by different pathogens (e.g., [3,5–7]). Nevertheless, depending on the mechanisms involved and which, if any, of the pathogens is competitively dominant, this phenomenon may severely affect the outcome of prophylactic or vaccination campaigns. If one pathogenic species is less sensitive to treatment but at a competitive disadvantage against the most sensitive one, this may lead to the opening of the widest gate to the most pathogenic microbe. It is thus essential to better understand the origin and mechanisms of such competitive interactions, not only for babesioses, theilerioses, and anaplasmoses that represent a real threat to livestock on a global scale, particularly in developing countries where they constrain economic improvement [8–10], but also for other vector-borne or other diseases affecting animals or humans. This parameter is thus worth investigating further, because it may open new perspectives in the design of therapeutic strategies of economically and medically important pathogenic agents, particularly in tick-borne and vector-borne diseases, where it has attracted little attention so far.

## Supporting Information

Figure S1 A Typical Landscape of El-Tarf Wilaya around Boutheldja, on the Northeast Border of Algeria (Close to Tunisia), East of Annaba in Algeria Several local breed cattle, as those studied in the present study, can be seen. All cattle belong to the so-called Atlas brown breed. The Cheurfa and Guelmoise sub-breeds harbor various clear coat colors, almost white for some. The Sétifienne displays a uniform black coat and the Chelefienne a fawn coat. Photo credit: Loubna Dib.
(2.8 MB TIF)
