# Supplementary figures and images for: Competitive Exclusion between Piroplasmosis and Anaplasmosis Agents within Cattle
Source: PLoS Pathog. 2008 Jan 25;4(1):e7. doi: 10.1371/journal.ppat.0040007 (PMC2323288; doi:10.1371/journal.ppat.0040007)

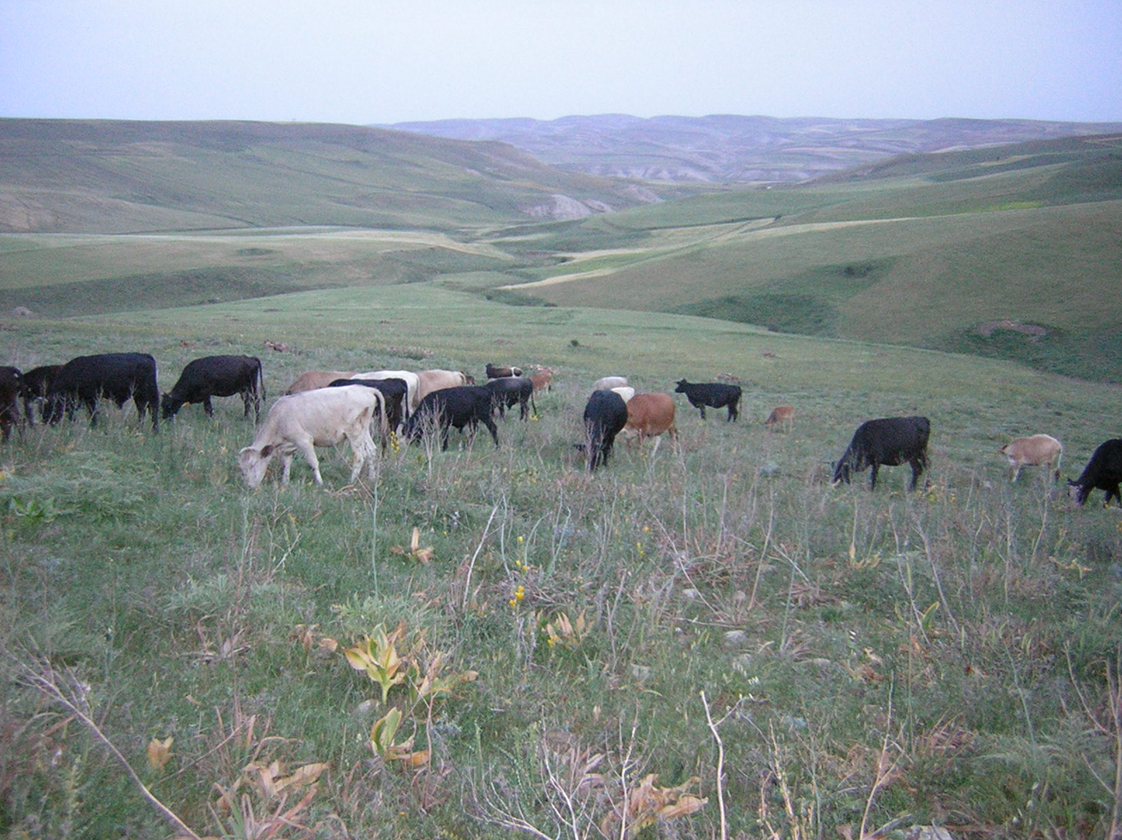

Supplement: Figure S1 — Several local breed cattle, as those studied in the present study, can be seen. All cattle belong to the so-called Atlas brown breed. The Cheurfa and Guelmoise sub-breeds harbor various clear coat colors, almost white for some. The Sétifienne displays a uniform black coat and the Chelefienne a fawn coat. Photo credit: Loubna Dib. (2.8 MB TIF) [file ppat.0040007.sg001.tif]
